# Association between depression, anxiety, and stress and oral health-related quality of life in adolescents

**DOI:** 10.1590/1807-3107bor-2025.vol39.109

**Published:** 2025-10-10

**Authors:** Eduarda da Silveira BORSTMANN, Bruna BRONDANI, Jessica Klöckner KNORST, Bruna Dal Pizzol SIQUEIRA, Luiza Saraiva de LIMA, Yassmín Hêllwaht RAMADAN, Bruno EMMANUELLI, Thiago Machado ARDENGHI

**Affiliations:** (a) Universidade Federal de Santa Maria – UFSM, School of Dentistry, Department of Stomatology, Santa Maria, RS, Brazil.

**Keywords:** Depression, Anxiety, Adolescent, Oral Health

## Abstract

This cross-sectional study aimed to evaluate the association between the degree of depression, anxiety, and stress and oral health-related quality of life (OHRQoL) of adolescents aged 11 to 19 years from Santa Maria, Brazil. The Child Perception Questionnaire (CPQ 11-14) was used to evaluate OHRQoL. The degree of anxiety, depression, and stress was assessed using the Depression, Anxiety and Stress Scale – Short Form (DASS-21). Additionally, sociodemographic, clinical, and psychosocial variables were considered. Adjusted Poisson regression models were applied to examine the associations between predictive variables and overall CPQ11-14 scores. Results are reported as rate ratio (RR) and 95% confidence intervals (95% CI). A total of 164 adolescents were evaluated. The mean DASS-21 scores for depression, anxiety, and stress were 6.9 (standard deviation [SD] 9.0), 8.7 (SD 8.9), and 11.9 (SD 9.7), respectively. The mean CPQ 11-14 score was 12.2 (SD 10.3). Adolescents with high levels of depression (RR = 1.03; 95%CI: 1.02–1.05) and stress (RR = 1.02; 95%CI: 1.01–1.04) presented higher CPQ 11-14 scores, indicating a significant impact of those symptoms on OHRQoL. In conclusion, adolescents with high depression and stress scores experienced poorer OHRQoL, highlighting the need for integrated mental health and oral health interventions to improve their overall well-being.

## Introduction

Adolescence is a critical period of transition from childhood to adulthood, marked by the pursuit of autonomy and by biological, behavioral, and psychosocial changes.^1,2^ Due to these changes, emotional disorders such as anxiety and depression are known to be increasingly present in this age group.^3^ According to the World Health Organization (WHO), around 14% of adolescents (between 10 and 19 years of age) have some mental disorder, but the majority of these are undiagnosed or undertreated.^4^ The behavioral and social changes that occur during this transition also contribute to changes in oral health.

Some studies have shown that oral injuries such as tooth decay and periodontal disease can affect well-being and consequently cause mental disorders, dental fear, and greater resistance during the clinical procedure, which lead to poorer oral conditions.^5-9^ Mental health conditions such as depression, anxiety, and stress can influence adolescents’ behaviors, including oral hygiene and their willingness to seek dental care.^5-9^ These psychological factors may contribute to a poorer oral health-related quality of life (OHRQoL).^5-9^ OHRQoL is a multidimensional construct used to measure the impact of oral conditions on daily activities and well-being of individuals.^10^ It includes aspects such as pain, functional limitations, self-esteem, and the impact of oral health on social interactions.^10^ Thus, anxiety, stress, and depression can significantly impact innumerable aspects of adolescents’ lives and their overall well-being,^11-15^ including OHRQoL.

Previous literature has shown the association between anxiety and depression and general quality of life considering other health areas.^13-15^. No previous study has specifically evaluated the association between depression, anxiety, and stress levels and OHRQoL in adolescents, highlighting the relevance of our research. Understanding this association is essential for clinicians as it highlights the need for an integrated approach to adolescent healthcare. Early identification of psychological distress could help dentists and other healthcare providers implement preventive strategies, improve adherence to oral health treatments, and enhance overall well-being. Interdisciplinary interventions that address both mental and oral health could lead to more effective and comprehensive care for adolescents.

Understanding this association is particularly relevant during adolescence, a phase marked by significant biopsychosocial changes and considerable emotional and behavioral instability. This study aimed to evaluate the association between depression, anxiety, and stress and OHRQoL of adolescents. Our conceptual hypothesis is that adolescents with higher levels of anxiety, stress, and depression are more likely to experience poorer OHRQoL.

## Methods

### Ethical considerations

This study was approved by the Human Research Ethics Committee of the Federal University of Santa Maria (UFSM) (process No. 0144.0.243.000-10). All parents or guardians of the participants signed the Free and Informed Consent Form and the adolescents signed the Assent Form. Since data collection was conducted at the institution’s Adolescent Clinic, an official authorization from the institution was obtained as part of the Ethics Committee’s approval process.

### Study design and sample

This cross-sectional study was conducted with a convenience sample of adolescents aged 11 to 19 years who received treatment at the Adolescent Clinic of UFSM between August 2016 and December 2019. The clinic is located in Santa Maria, southern Brazil, and provides free multidisciplinary dental care to patients from the city and surrounding areas, serving an average of 50 adolescents per year. Treatments are performed by undergraduate students in their final years of training under the supervision of postgraduate students and faculty members. All participants attended public schools in the region and came from low-income families.

Adolescents who sought evaluation or dental care were assisted and included in the research. Patients who had any diagnosed physical or psychological limitations were excluded. A post-hoc power analysis was conducted using G*Power software based on the following parameters: a significance level of 0.05, an effect size of 0.3 (considered a medium effect), a sample size of 164 participants, and a regression model with 8 predictors. The results indicated that the study had a power of 100% to detect differences, suggesting that the sample size was adequate to identify meaningful effects.

### Oral Health-Related Quality of Life

To assess the OHRQoL of participants, we administered the Brazilian short version of the Child Perceptions Questionnaire for 11- to 14-year-olds (CPQ11-14) through face-to-face interviews conducted by trained interviewers prior to dental treatment.^16,17^ This questionnaire has 16 items that evaluate the frequency of events related to oral conditions and is divided into four domains: oral symptoms, functional limitations, emotional well-being, and social well-being. Response options are scored as follows: “Never” = 0; “Once or twice” = 1; “Sometimes” = 2; “Often” = 3; and “Every day/almost every day” = 4. The overall score is obtained by summing the individual item scores, ranging from 0 to 64, where higher scores indicate a greater negative impact of oral conditions on OHRQoL.^18^

### Depression, anxiety, and stress

Our main predictors, depression, anxiety and stress, were assessed with the Depression, Anxiety and Stress Scale – Short Form (DASS-21), developed by Lovibond and Lovibond^12^ to measure and differentiate the symptoms of anxiety, depression and stress as much as possible. The version used in this study was an adaptation of the version for Brazilian adults by Machado and Bandeira.^19^ Similar to the CPQ11-14, the DASS-21 was administered before dental care by trained interviewers. This instrument consists of 21 questions equally subdivided into 3 factors across 3 scales - depression, anxiety, and stress. Answers range from 0 to 3: “Not applied at all” = 0, “Applied to some degree, or for a short time” = 1, “Applied to a considerable degree, or for a good part of the time” = 2, and “Applied a lot, or most of the time” = 3. Scores for depression, anxiety, and stress are calculated by summing the scores for the corresponding items and multiplying by 2 to calculate the final score. The higher the score for each scale, the greater the severity of the respective factor.

### Confounders variables

Adolescents also completed a structured questionnaire addressing sociodemographic and psychosocial characteristics. Information regarding sex (“girls” and “boys”) and household family income was collected. Household income, recorded in Brazilian real (with an exchange rate of approximately 5.44 reals to 1 US dollar), encompassed the total income of all household members—including salaries, Bolsa Família benefits, pensions, retirements, and other income sources—and was used as a continuous quantitative variable in the statistical analysis. Psychosocial variables included self-perceived need for dental treatment (“yes” or “no”) and dental fear. To assess dental fear, adolescents rated their level of fear regarding dental visits using the following options: “Not at all” = 0, “A little” = 1, and “A lot” = 2.

Regarding oral health status, two characteristics were considered –toothache and dental caries. Toothache was assessed considering the last six months with the following answer options: “No” = 0, “Yes” = 1, and “I don’t remember” = 2. Dental caries was assessed through visual inspection with the aid of a flat dental mirror and periodontal probes (CPI; “ball point”), using the International Caries and Detection Assessment System (ICDAS).^20^ Clinical examinations were carried out in dental chairs with the aid of artificial light after dental prophylaxis by previously trained and supervised examiners. For statistical analysis, surfaces were categorized into carious (scores 1, 2, 3, 4, 5, and 6) and non-carious surfaces (score 0).

### Statistical analysis

Data analysis was conducted with STATA 14.0 (Stata Corporation, College Station, USA). A descriptive analysis of general sample characteristics was performed. The outcome of the study was overall CPQ11-14 scores. Pearson correlation was performed to assess the relationships between anxiety, stress, and depression. Additionally, the variance inflation factor (VIF) was calculated to check for multicollinearity. VIF values greater than 10 suggest significant multicollinearity, which could affect the validity of the regression coefficients.

Adjusted Poisson regression models were used to evaluate the relationship of anxiety, depression, and stress with OHRQoL. Confounding variables were selected based on the theoretical framework of the model, acknowledging that psychosocial factors are influenced by the broader structural and contextual conditions in which they are embedded.^21^ To support the identification of true confounders, we constructed a DAG (Figure), which served as the basis for the inclusion of variables in the multivariable models. All variables with a p-value < 0.20 in the unadjusted analysis were retained in the adjusted model, which was developed using a stepwise forward approach. We assessed the adequacy of the Poisson model by testing for overdispersion through the deviance-to-degrees-of-freedom ratio. As the ratio was below the conventional threshold (1.35), the assumption of equidispersion was accepted. Nevertheless, we also conducted sensitivity analyses using negative binomial regression models, which yielded similar estimates, confirming the robustness of our findings. The results are presented as rate ratios (RR) with corresponding 95% confidence intervals (CIs). Variables with p-values < 0.05 in the final model were considered statistically significant.

**Figure 1:**
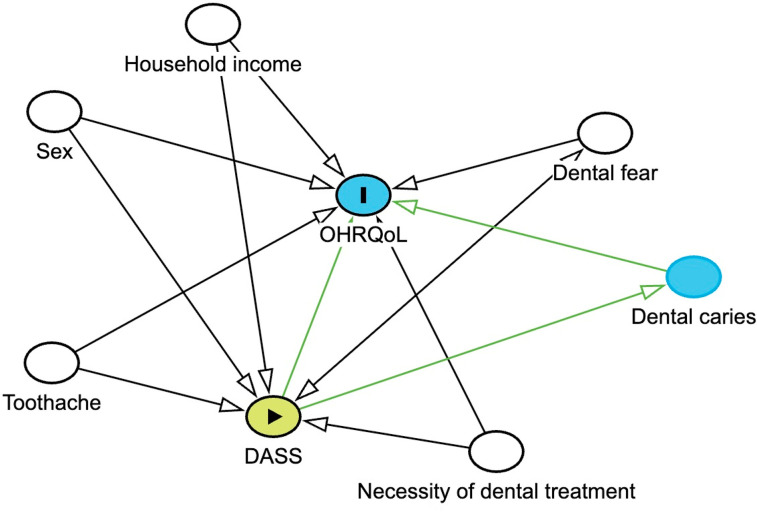
Directed acyclic graph (DAG) with a conceptual representation among the relationship DASS, OHRQoL and possible confounders factors DASS, Depression, Anxiety and Stress Scale - 21 Items; OHRQoL, oral health-related quality of life; (⏵) Main predictor; (▎) Outcome; ⬭ Possible confounders.

## Results

A total of 164 adolescents were included in the study. As shown in Table 1, the participants were equally divided between boys and girls (50%) and reported an average monthly family income of R$1,845.00 [standard deviation (SD) 1,225.00]. Among the respondents, 48.1% reported having had toothache and 7.8% perceived the need for dental treatment. In addition, 47.8% of adolescents reported feeling at least a little fear of visiting the dentist, while 95.1% had dental caries. Regarding the psychosocial questionnaires, participants had an average score of 6.9 (SD 9.0), 8.7 (SD 8.9), and 11.9 (SD 9.7) for the depression, anxiety, and stress DASS scales, respectively, and a mean CPQ11-14 score of 12.2 (SD 10.3).

**Table 1:** Sociodemographic characteristics and oral health measures of evaluated adolescents (n = 164).

Variables	n (%)	Total
Sex		164
Girls	82 (50.0)	
Boys	82 (50.0)	
Toothache		52
No	25 (48.1)	
Yes	27 (51.9)	
Self-perception of need for dental treatment		51
No	47 (92.2)	
Yes	4 (7.8)	
Dental fear		44
None	23 (52.2)	
A little bit	16 (36.4)	
A lot	5 (11.4)	
Dental caries		123
Without	6 (4.9)	
With	117 (95.1)	
	Mean (SD^a^)	Total
DASS-depression	6.90 (9.00)	164
DASS-anxiety	8.73 (8.92)	164
DASS-stress	11.95 (9.75)	164
Household income	1845 (1225)	128
OHRQoL (overall CPQ11-14 score)	12.16 (10.31)	164

^a^Standard deviation, SD; *Values less than 164 are due to missing data; DASS: Depression, Anxiety and Stress Scale OHRQoL: oral health-related quality of life.

Table 2 shows the results of the unadjusted analysis. All evaluated factors were significantly associated with CPQ11-14 scores (p < 0.05). Male individuals had CPQ11-14 scores 19% lower than their counterparts. Individuals from families with higher monthly incomes presented lower CPQ11-14 scores. In addition, adolescents who reported having experienced a toothache and perceived the need for dental treatment had CPQ11-14 scores 54% and 38% higher, indicating poorer OHRQo. Furthermore, participants who reported fear of going to the dentist presented OHRQoL scores 2.63 times higher than those without dental fear. Regarding the depression, anxiety, and stress scales, adolescents with higher scores on the DASS had a 3 to 4% higher prevalence of poorer OHRQoL.

**Table 2:** Unadjusted analysis of the association between predictive variables and OHRQoL using Poisson Regression analysis.

Variables	Unadjusted	p-value
	RR^a^ (95%CI^b^)	
Sex		< 0.01
Girls	1	
Boys	0.81 (0.74–0.88)	
Household income	0.99 (0.99–0.99)	< 0.01
Toothache		< 0.01
No	1	
Yes	1.54 (1.33–1.78)	
Self-perception of need for dental treatment		< 0.05
No	1	
Yes	1.38 (1.02–1.87)	
Dental fear		< 0.01
None	1	
A little bit	1.41 (1.18–1.68)	
A lot	2.63 (2.15–3.23)	
DASS-depression	1.04 (1.03–1.04)	< 0.01
DASS-anxiety	1.04 (1.03–1.04)	< 0.01
DASS-stress	1.03 (1.02–1.03)	< 0.01

PR: Rate ratio; CI: confidence interval; DASS: Depression, Anxiety and Stress Scale; OHRQoL: oral health-related quality of life.

The adjusted analysis between predictors and OHRQoL is shown in Table 3. Adolescents with higher scores on the depression and stress DASS scales exhibited 3% (RR = 1.03; 95%CI: 1.02–1.05) and 2% (RR = 1.02; 95%CI: 1.01–1.04) higher scores in CPQ11-14, indicating poorer OHRQoL. Adolescents who reported mild and severe fear of going to the dentist also presented poorer OHRQoL, with overall CPQ11-14 scores of 79% (RR = 1.79 95%CI: 1.38–2.33) and 70% (RR = 1.70 95%CI: 1.26–2.30) higher, respectively, than those without fear.

**Table 3:** Adjusted analysis of the association between predictive variables and OHRQoL determined by Poisson Regression analysis.

Variables	Adjusted	p-value
	RR^a^ (95%CI^b^)	
Sex		0.12
Girls	1	
Boys	0.84 (0.68–1.04)	
Household income	0.99 (0.99–1.01)	0.23
Toothache		0.82
No	1	
Yes	1.03 (0.79–1.34)	
Self-perception of need for dental treatment		0.32
No	1	
Yes	1.26 (0.79–2.01)	
Dental fear		< 0.01
None	1	
A little bit	1.79 (1.38–2.33)	
A lot	1.70 (1.26–2.30)	
DASS-depression	1.03 (1.02–1.05)	< 0.01
DASS-anxiety	0.98 (0.96–1.01)	0.27
DASS-stress	1.02 (1.01–1.04)	< 0.05

PR: Rate ratio; CI: Confidence interval; DASS: Depression, Anxiety and Stress Scale; OHRQoL: oral health-related quality of life.

Correlation analysis showed that anxiety, depression, and stress were significantly correlated (p < 0.05). However, the collinearity test showed VIF values ranging from 2 to 3, indicating no multicollinearity in the model.

## Discussion

This study aimed to evaluate the association between depression, anxiety, and stress levels and the OHRQoL of adolescents. To the best of our knowledge, this is the first study to investigate these associations in this population. Our findings partially confirmed the initial hypothesis, as higher levels of depression and stress, as measured by the DASS scale, were associated with poorer OHRQoL. However, anxiety did not show a significant association with OHRQoL in this group. These results highlight the need for further research on psychosocial factors of adolescence, as they play a fundamental role in this critical developmental stage and may have lasting effects on overall health and well-being in adulthood.

The association between depression and poorer OHRQoL observed in this study is consistent with previous research across different age groups.^22-24^ This relationship may be explained by the well-documented link between low self-esteem and depression,^25,26^ which often intensifies concerns about physical appearance, including dental aesthetics. Feelings of shame regarding oral health are common in adolescents experiencing depression and are frequently accompanied by inferiority complexes. These emotions can negatively impact social interactions, undermining self-confidence and leading to social withdrawal.^27^ Additionally, the pervasive influence of social media during adolescence intensifies self-comparison, further lowering self-esteem.^28^ Collectively, these factors contribute to a decline in OHRQoL among adolescents.

Similar to depression, stress was significantly associated with poorer OHRQoL in our study. It is widely known that stress promotes unhealthy habits that can negatively impact oral health, ultimately leading to declines in both overall quality of life and OHRQoL.^29^ During stressful periods, individuals often turn to comfort foods, which are typically high in sugar and refined carbohydrates,^30^ increasing the risk of dental caries.^31^ Additionally, stress can trigger other maladaptive coping mechanisms, such as excessive alcohol consumption and smoking, both of which are well-established risk factors for periodontitis and oral cancer.^32^ Another notable consequence of stress is bruxism, a condition that can lead to tooth wear, jaw pain, and temporomandibular dysfunction (TMD).^33^ Previous studies have consistently demonstrated that bruxism negatively affects OHRQoL.^34^

In contrast to depression and stress, the association between anxiety and OHRQoL was not maintained in the adjusted analysis. It is important to emphasize that anxiety may be strongly correlated with behaviors like binge eating, which may lead to the consumption of high-carbohydrate and high-sugar foods, potentially impacting oral health.^35^ However, the effects of anxiety on OHRQoL may be less pronounced compared with other mental health variables in our sample. This may be due to the distinct nature of the complex psychosocial variables considered. Stress is often associated with unhealthy coping mechanisms such as poor dietary choices, smoking, and neglect of self-care, all of which can negatively affect perceived oral health.^29-34^ Depression is also often linked to lack of motivation and self-care, which may contribute to a decline in OHRQoL.^25,28^ These findings highlight the importance of considering the complex interplay of different psychological factors when assessing their impact on oral health. Future research should explore these relationships in more detail.

Our findings also indicate that fear of visiting the dentist is often mistaken for the anxiety, depression, and stress levels assessed with the questionnaire. Dental fear and anxiety, particularly before appointments, are common among children and adolescents and have long been a challenge for professionals.^36^ In our study, fear of visiting the dentist was associated with poorer OHRQoL, reinforcing its impact on oral health perception. This result is consistent with a previous study conducted in Finland with children aged 11 to 14 years, which highlighted the negative effects of dental fear on OHRQoL, particularly in emotional and social well-being. The authors suggest that fear of dental procedures may lead individuals to feel inferior to their peers or ashamed of their inability to cope with treatment.^37^ As a result, this can diminish self-confidence and self-esteem, further impairing OHRQoL.

Several limitations of this study should be acknowledged. First, its cross-sectional design limits causal inferences regarding the findings. However, it is important to highlight that no previous study has explored the relationship between depression, anxiety, stress, and OHRQoL in adolescents. Therefore, we recommend that future research adopt longitudinal designs to provide a deeper understanding of how these psychosocial factors influence individuals’ quality of life over time. Additionally, this study relied on a convenience sample, which may lead to selection bias and reduced representativeness of the target population. This sampling approach was chosen due to the accessibility and willingness of the adolescents to participate in the study. As our sample was restricted to individuals who attended a dental clinic, the findings may not be generalizable to those who do not seek dental services, which may lead to an underestimation of the association between mental disorders and barriers to dental care. Another limitation of this study is that medication use was not assessed. The use of medications, particularly those for managing stress, anxiety, and depression, could be an important confounding factor influencing the relationship between psychological factors and OHRQoL. While we recognize the study limitations, we believe that this approach allowed specific questions to be explored and contributed to a more comprehensive understanding of adolescents oral health needs. Nonetheless, future studies should consider a more representative sample and other confounding factors.

Our study has several strengths. First, the DASS-21 scale was used because it has a well-established adaptation and validation, making it a reliable tool for assessing and clearly measuring symptoms of depression, anxiety, and stress^12^. This scale has demonstrated strong stability and has been translated and validated for various age groups and across different countries,^38^ ensuring its broad applicability. Additionally, the CPQ11-14 was used to evaluate the OHRQoL, which further reinforces the study’s focus on the adolescent population. Our findings underscore the need to address mental health concerns alongside oral health, as both are vital components of overall well-being, particularly in the context of adolescent dental care.

## Conclusion

Adolescents with elevated levels of depression and stress have a significantly poorer OHRQoL than their peers without these psychological challenges. These findings highlight the crucial role of emotional and social factors in the assessment of oral health, particularly during the critical developmental period of adolescence.

## Data Availability

The datasets generated during and/or analyzed during the current study are available from the corresponding author on reasonable request.
